# Students’ satisfaction with general practitioners’ feedback to their reflective writing: a randomized controlled trial

**Published:** 2017-12-15

**Authors:** Alexander Kiss, Claudia Steiner, Paul Grossman, Wolf Langewitz, Peter Tschudi, Claudia Kiessling

**Affiliations:** 1 Department of Psychosomatic Medicine, University Hospital Basel, Basel, Switzerland; 2 Institute of General Practice, University of Basel, Basel, Switzerland; 3 Munich University Hospital, Ludwig-Maximilians-University, Munich, Germany; 4 Brandenburg Medical School, Neuruppin, Germany

## Abstract

**Background:**

Reflective Writing (RW) is increasingly being implemented in medical education. Feedback to students’ reflective writing (RW) is essential, but resources for individualized feedback often lack. We aimed to determine whether general practitioners (GPs) teaching students clinical skills could also provide feedback to RW and whether an instruction letter specific to RW feedback increases students’ satisfaction.

**Methods:**

GPs were randomized to the two study arms using block randomization. GPs in both groups received an instruction letter on giving students feedback on clinical skills. Additionally, intervention group GPs received specific instructions on providing feedback to students’ RW. Students completed satisfaction questionnaires on feedback received on clinical skills and RW. T-tests were employed for all statistical analysis to compare groups.

**Results:**

Eighty-three out of 134 physicians participated: 38 were randomized to the control, 45 to the intervention group. Students were very satisfied with the feedback on RW and clinical skills regardless of tutors’ group allocation. A specific instruction letter had no additional effect on students’ satisfaction.

**Conclusion:**

Based on student satisfaction, GPs who give students feedback on clinical skills are also well suited to provide feedback on RW. This approach can facilitate the introduction of mandatory RW into the regular medical curriculum.

## Introduction

Reflection is essential to learning from experience and an integral part of the professional formation of medical students.^1^ To promote self-reflection, empathy, and reflection upon practice, Reflective Writing (RW) programs are increasingly being implemented at medical schools.^1^ How RW is performed and evaluated depends on the goal addressed. Charon^2^ suggests that writing itself teaches the skills of reflection. Therefore, teachers do not rate, but provide feedback, after close reading of the text. If the goal is to guide structured feedback to students’ RW, The Brown Educational Guide to the Analysis of Narrative (BEGAN)^3^,^4^ is recommended. To assess students’ reflective capacity for relationship with later academic achievement, a modified version of BEGAN without structured feedback is useful.^5^

Formative feedback to foster professional development^1^,^3^,^6^ is considered as essential for developing reflective capacity in students as it is in traditional clinical learning.^7^ A recent national (USA) survey of RW programs showed that roughly three-fourths of the institutions with such programs used individual student feedback as a teaching strategy.^1^ Students’ satisfaction with the feedback, however, has never been investigated systematically.

Additional faculty time and energy required to review and provide meaningful individual feedback on numerous reflective papers may hamper or prevent implementation of RW, especially if mandatory. We wanted to determine whether it was feasible that general practitioners (GPs), versed in giving feedback on medical students’ clinical skills, could also provide satisfactory RW feedback.

In Basel (Switzerland), all fourth-year medical students actively participate one half-day per week over two semesters in one-on-one tutorials in a GP’s office.^8^ The Medical Faculty and Institute of General Practice, co-responsible for these tutorials, agreed to pilot mandatory students’ RW with GPs’ feedback and to study students’ satisfaction thereof. The hypotheses were: 1) GPs could provide satisfactory RW feedback; and 2) an instruction letter specific to RW feedback would increase students’ satisfaction.

## Methods

### Study design

This study was a randomized control trial in which the students were blinded to the intervention.

### General practitioners

All GPs partaking in one-on-one tutorials in 2011 had been trained in a mandatory half-day seminar to teach and provide feedback on students’ clinical skills. They were invited to participate in the study. Physicians who consented were randomized into two groups. PG, who was fully blinded to GP identity except GP study identification number, conducted randomization employing a random-event generator (www.randomizer.org), using blocks of 4–6. Both control and intervention groups received a letter on providing clinical skills feedback. Additionally, the intervention group received specific instructions on how to provide feedback on RW, adapted from and closely followed the framework presented by Reis^3^ (see Table 1 in Appendix A).

### Students

All fourth-year students completed their mandatory RW assignments during the first six-months of the tutorial. All students were asked to write about and reflect upon two interactions with different patients encountered in the GP’s office - a remarkable encounter with a patient and an encounter with a patient for whom they felt little or no empathy. Students were unaware of the GP intervention.

### Reflective writing

Students received written instructions to increase observational acuity and description accuracy (patient description, first impressions, interaction with patient, etc.). Prompts were provided to encourage reflection (e.g., “What made me feel little or no empathy for this particular patient?” and “What was special about the patient or my behavior?”).^9^ Students were asked to reflect on the impact the encounters could have on their professional behavior with future patients.

After giving their RW essays to their personal tutors for reading and receiving oral feedback, students filled out a written satisfaction questionnaire consisting of two parts: A) Six items on satisfaction with feedback on clinical skills (from the validated questionnaire of the Swiss National Survey of Residents’ Training Programs^10^); and B) eight items on satisfaction with feedback on RW (adapted from Reis et al.^3^ as there is no validated questionnaire assessing students’ satisfaction with tutors’ RW feedback) (see Table 2 in Appendix A). All students electronically submitted their essays and satisfaction questionnaires to AK, responsible for the Medical Humanities Curriculum and principal investigator. RW essays were not graded and tutors had no access to the satisfaction questionnaires.

### Data analysis

T-tests were used for group comparisons of the sum scores of each of the two scales (p< 0.05). Individual items were also analyzed, employing Bonferroni adjustments for multiple comparisons. Association between scale sum scores was evaluated by Pearson correlation. All analyses were conducted using Statistica 10 (StatSoft Inc.).

### Ethics statement

This study was approved by the Ethics Committee of Both Basels. The anonymity of the general practitioners and students was guaranteed.

## Results

Eighty-three out of 134 GP tutors participated in the study. Forty-five were randomized to the intervention and 38 to the control group.

T-tests indicated that the intervention group GPs did not differ from control group GPs in age (53.07, SD 6.86 *vs.* 53.08, SD 6.84), gender (female 7, 15.5% *vs.* 9, 23%), years since primary qualification (27.11, SD 7.15 *vs.* 26.00, SD 5.96) or years in general practice (18.41, SD 7.78 *vs.* 15.90, SD 7.83).

All students (N=83, f=50, m=33, aged 24.5, SD 2.06) completed their RW and filled out the satisfaction questionnaires. Data were normally distributed. The *t*-tests revealed no differences on any student demographic between the intervention or control group. No differences were found for the sum scores of the two scales (clinical skills t(81) =0.78, p=.44, RW t(81)=-0.03, p=0.98) or the individual questions after Bonferroni adjustments. Students were highly satisfied with feedback to their clinical skills and their RW essays regardless of group allocation (Table 2). Satisfaction with feedback to clinical skills was positively correlated with satisfaction with specific feedback to RW (r=0.56, P<0.0001).

## Discussion

### Main findings

Medical students were very satisfied with the feedback on RW and general feedback on clinical skills. High satisfaction with clinical skills feedback was associated with high satisfaction with RW feedback. Specific RW feedback instructions to tutors had no additional effect on students’ satisfaction.

Medical students’ satisfaction with the clinical skills feedback from GP tutors corresponds to previous research.^8^ Isler et al. reported that one-on-one tutorials in Basel received an overall rating of 5.3 on a 6-point scale, the highest rank among all university medical faculty classes. Students concurred that they had increased their skills and knowledge, acquired social and communicative skills, encountered a varied patient population, and reported good personal relationships with their tutors. Our findings corroborate these results and show that satisfaction with tutor feedback on clinical skills is rated far better than residents’ satisfaction with feedback in clinical settings as reported in the Swiss national survey.^10^

Several explanations as to why students value their tutors’ RW feedback so highly, regardless of whether the GP followed specific instructions, are plausible. Trained in giving general clinical feedback, tutors may have applied these skills to RW feedback. Students and tutors establish personal relationships with tutors serving as role models, helping the students improve their skills and knowledge, and assisting them in developing their own professional identities. The overall satisfaction with the one-on-one tutorials in GP practices may generalize to the feedback received to their RW. The positive correlation between satisfaction to feedback on clinical skills and satisfaction with feedback on RW supports this explanation. Furthermore, a ceiling effect may explain the lack of effect of specific tutor instructions.

Whereas tutors with non-GP backgrounds may encounter difficulties finding acceptance and getting their messages across,^11^ students accepted and appreciated GPs’ feedback to RW. The GP has the advantage of knowing both student and patient, which can be important, e.g., when clarifying how to maintain a professional demeanor with a patient for whom the student felt little or no empathy (see Table 2 in Appendix A).

This study has several limitations. The personal relationship between tutor and student and giving the two questionnaires simultaneously may have potentially added a confounding bias to the scores. Despite high student satisfaction, we do not know whether the GPs read and applied the feedback instructions, how the feedback was performed, or whether it enhanced students’ self-reflection and reflection upon practice.^12^,^13^. Reflection has not been assessed. Further research should focus on how GPs give feedback and assess students’ reflective capacity in RW.^2^,^4^,^14^

In this pilot study accompanying the introduction of RW in the medical curriculum, self-rated satisfaction with tutor feedback represents a first step. Koepke^11^ and Aronson et al.^12^ point out that resistance to adding reflective tasks, particularly written reflection to a full curriculum, can be an issue for both students and staff. Satisfaction is seen as a measure of students’ acceptance of GPs’ feedback to this novel assignment and indirectly, GPs’ acceptance of providing feedback.

### Conclusion

Our pilot study shows that distributing the sizeable task of providing feedback to mandatory RW assignments among a large number of GPs involved in teaching clinical skills to medical students is feasible. This can facilitate the introduction of RW into the medical curriculum. Medical schools that involve GPs in student training may want to consider drawing on this population of professionals to implement RW in the curriculum.

## Appendix A

**Table 1:** Tutor instruction for oral feedback to students’ reflective writing texts*

Before the feedback
Gratefully acknowledge receiving the text or remind the student once if the deadline has passed. There is nothing administrative (e.g. sign, confirm) that you have to do! Read the text for a first time without a pen. The aim of your first reading is your overall impression. Reread the text – this time with pen and paper - Mark the passages in the text: Description of the situation. - Mark the passages in the text: Thoughts, how the situation arose. - Mark the passages in the text: Lessons learned. Close reading approach: Provide feedback only on the items that seem meaningful to you and with which you feel confident. - How precisely does the student describe the situation / his or her reflections / the lessons learned - Use of imagery, for example “Then the patient blew his top” - Use of metaphors: e.g. “I take my hat off to someone with such fighting spirit “ - Is there a plot? Example: detective story reduced to its essence: “A corpse is found. The detective tries to find the murderer.” - Is there conflict in the plot? - Differences between the patient’s and the student’s concept. Prepare reflection-inviting questions: - What was the patient thinking? - Why did the patient act this way? - How did the student feel about it? - Are observation, reflection, and lessons learned coherent or not? - Might there be alternative explanations? Make an appointment with the student for your oral feedback.
**Feedback**
Say how much time you have and that you read the text. Start with what you found positive about the text: Specific feedback on concrete aspects *(What was good and why!)* If there was something in the text that you did not understand: Ask nicely! Ask questions that encourage reflection and discussion with the student. If you find shortcomings in the text your line of reasoning should be comprehensible to the student. Make concrete suggestions for improvement. Address the points that came to your attention when you read the text closely. For the text “The patient that impressed you in particular“ provide feedback on how the student describes, terms *(“I tried to reflect the patients feelings”)* and reflects on the communication techniques learned in the „patient interview communication skills course“. Provide more extensive feedback to the text “The patient, for whom I felt little/no empathy” and initiate a discussion: You know the patient too and have more or less empathy for him or her. Discuss what maintaining a professional demeanour when dealing with such a patient involves. Use your “Instructions for oral feedback” as a checklist to ensure that all points are covered.

* Based on and adapted from Reis et al.^3^

**Table 2:** Students’ satisfaction with feedback on clinical skills and reflective writing Satisfaction with Feedback*

to Clinical Skills†	Intervention Group Mean (SD) N=45	Control Group Mean (SD) N=38
I regularly receive feedback during my tutorial on what I do right and what I do incorrectly.	5.0 (1.02)	5.0 (1.17)
In addition to general feedback (e.g. “this anamnesis was well done”), I also receive feedback on specific and particular aspects of my work (what exactly was good about the status or the case history etc.)	4.8 (1.25)	4.8 (1.10)
If shortcomings are discerned, feedback includes a plan with specific steps for improvement.	4.8 (1.20)	4.7 (1.16)
I receive feedback on my clinical work on and with the patient: anamnesis, clinical assessment, patient briefing etc.	4.9 (1.18)	5.1 (1.02)
I receive feedback on my practical technical work on and with the patient: measuring blood pressure, injections, manual skills, etc.	5.2 (1.06)	5.3 (1.08)
I receive feedback on my practical clinical work without patients: case presentations, reports, etc.	4.2 (1.46)	4.8 1.13)
Sum scores	40.5 (8.14)	40.5 (5.58)
to Reflective Writing§		
I received feedback from my tutor on my reflective writing assignment.	5.6 (.92)	5.7 (.66)
I have the impression that my tutor read my text carefully (for example by citing text passages).	5.5 (.94)	5.6 (.69)
In addition to receiving general feedback (e.g. “it was a good text”), I received feedback on specific aspects (what was good and why).	5.2 (1.17)	5.0 (1.04)
If my tutor found shortcomings in my text, the line of reasoning was comprehensible and concrete suggestions for improvement were made.	5.1 (1.14)	5.0 (1.05)
My tutor’s questions encouraged me to reflect.	4.8 (1.51)	4.7 (1.19)
My tutor’s feedback on my text concerning “a remarkable encounter” helped me reflect on the communication techniques learned in the “communication skills course”.	4.4 (1.40)	4.3 (1.47)
My tutor’s feedback on my text concerning “The patient for whom I felt little/no empathy” helped me better understand how this special interaction came about.	4.7 (1.62)	5.0 (.77)
The discussion with my tutor about my text “The patient for whom I felt little/no empathy” helped clarify what maintaining a professional demeanor when dealing with such a patient involves.	5.2 (1.23)	5.3 (.97)
Sum scores	28.9 (6.03)	29.8 (5.38)

* Items were assessed on a six-point scale ranging from 1 “does not apply at all” to 6 “fully applies.”

† Adapted from Van der Horst K et al 2010 10

§ Adapted from Reis et al 2010 3
